# Real-time monitoring of extracellular ATP in bacterial cultures using thermostable luciferase

**DOI:** 10.1371/journal.pone.0244200

**Published:** 2021-01-22

**Authors:** Julian Ihssen, Nina Jovanovic, Teja Sirec, Urs Spitz

**Affiliations:** 1 Biosynth AG, Staad, Switzerland; 2 Faculty of Biology, Department of Biochemistry and Molecular Biology, Institute of Physiology and Biochemistry, University of Belgrade, Belgrade, Serbia; 3 Carbosynth Limited, Axis House, Compton, Berkshire, United Kingdom; Cornell University, UNITED STATES

## Abstract

Adenosine triphosphate (ATP) is one of the most important indicators of cell viability. Extracellular ATP (eATP) is commonly detected in cultures of both eukaryotic and prokaryotic cells but is not the focus of current scientific research. Although ATP release has traditionally been considered to mainly occur as a consequence of cell destruction, current evidence indicates that ATP leakage also occurs during the growth phase of diverse bacterial species and may play an important role in bacterial physiology. ATP can be conveniently measured with high sensitivity in luciferase-based bioluminescence assays. However, wild-type luciferases suffer from low stability, which limit their use. Here we demonstrate that an engineered, thermostable luciferase is suitable for real-time monitoring of ATP release by bacteria, both in broth culture and on agar surfaces. Different bacterial species show distinct patterns of eATP accumulation and decline. Real-time monitoring of eATP allows for the estimation of viable cell number by relating luminescence onset time to initial cell concentration. Furthermore, the method is able to rapidly detect the effect of antibiotics on bacterial cultures as Ampicillin sensitive strains challenged with beta lactam antibiotics showed strongly increased accumulation of eATP even in the absence of growth, as determined by optical density. Patterns of eATP determined by real-time luminescence measurement could be used to infer the minimal inhibitory concentration of Ampicillin. Compared to conventional antibiotic susceptibility testing, the method presented here is faster and more sensitive, which is essential for better treatment outcomes and reducing the risk of inducing antibiotic resistance. Real-time eATP bioluminescence assays are suitable for different cell types, either prokaryotic or eukaryotic, thus, permitting their application in diverse fields of research. It can be used for example in the study of the role of eATP in physiology and pathophysiology, for monitoring microbial contamination or for antimicrobial susceptibility testing in clinical diagnostics.

## Introduction

### Role of extracellular ATP (eATP) in prokaryotic and eukaryotic cells

Adenosine triphosphate (ATP) is the universal energy carrier in all living systems mediating intracellular energy transfer, and enabling cells to store and transport chemical energy through its high-energy phosphate bonds [1]. In addition to the intracellular metabolic functions of ATP that are necessary for survival, growth and replication of cells, numerous roles for extracellular ATP (eATP) have been reported such as signalling, including interbacterial and host-bacteria communication, immune system and neuromodulation, pain, and pathophysiology [2–5]. While it has long been known that rapid release of ATP occurs during cell death, live bacteria have also been found to release ATP during their growth phase, including commensal bacteria in the gastrointestinal tract [2]. Bacterial eATP in the gut elicits a variety of inflammatory responses and interacts with the host’s mucosal immune system [3]. For example, intestinal ATP increases the number of Th17 cells in the gut [6], which is associated with the pathogenesis of inflammatory bowel disease (IBD) [7]. Similarly, ATP release has been observed in eukaryotic cells including neuronal and immune cells, as well as in injured or damaged cells [4]. Several studies have reported that eukaryotic cells release ATP via exocytosis, ATP-containing granules, plasma membrane carriers or large conductance channels [8–11]. These findings suggest that eATP plays important roles in both bacterial and human physiology, and operates as a regulatory signal in cross-talk between bacteria and host, which in turn regulates the bacterial community and stabilizes the gut ecosystem [6]. Moreover, eATP may play a role in interbacterial signalling since its extracellular concentration varies with the growth state of bacteria [2]. Release of ATP from bacterial cells may also be interpreted as an altruistic action since it might provide energy and nutrients for the neighbouring bacterial community. Addition of eATP to cultures was found to improve survival of *Escherichia coli* and *Salmonella* spp. in the stationary phase [2] supporting the claim for such altruistic actions.

### Role of eATP in bacterial physiology and pathogenesis

ATP leakage during the log growth phase in bacterial culture has been reported for diverse species including Gram positive and Gram negative bacteria [2, 12]. It was further proposed that the ATP release can be considered a common phenomenon in bacteria, while the dynamics of its release are species-specific [2]. In comparison to intracellular levels of ATP that range from 1 to 5 mM, extracellular concentrations are significantly lower, ranging from several nanomolar to several hundred nanomolar, representing 3–5% of the total ATP in bacterial culture [2]. Mempin and colleagues [2] recorded a peak eATP release during the late log phase to early stationary phase for most of the tested bacteria belonging to different families. The eATP levels then decreased to significantly lower or undetectable levels after 24h of growth. Interestingly, a depletion of eATP was also reported in cultures of *E*. *coli* and *Salmonella* spp., and in these cases the depletion was ostensibly caused by either eATP intake or by eATP degradation at the outer surface of the bacterial cells. In addition, glycolysis was found to be essential for ATP release [2] with glucose inducing ATP release dependant on the growth phase [12]. Moreover, ATP release was found to be dependent on cytochrome oxidases and respiration [2]. These findings support claims that bacterial cell death and lysis are not the only source of the extracellular ATP [2].

The role of eATP as a signalling molecule was also investigated in the processes of pathogenesis and biofilm formation. Extracellular ATP has been shown to enhance biofilm formation of nosocomial pathogens, namely *E*. *coli*, *Acinetobacter baumannii*, *Stenotrophomonas maltophilia*, and *Staphylococcus aureus* [13]. Furthermore, eATP was found to induce the dispersal of *F*. *nucleatum* biofilm which possesses distinct virulence characteristics and contributes to significantly higher production of pro-inflammatory cytokines compared to undetached biofilm and planktonic forms [14]. Endogenously produced eATP has been recognized as a signalling molecule that directs movement in twitching motility-mediated biofilm of *P*. *aeruginosa*, an opportunistic pathogen that infects damaged epithelial tissues. *P*. *aeruginosa* colony produces endogenous eATP at the edge of the actively expanding biofilm (≈3 mM), enabling a signal through a gradient of eATP where high concentrations of eATP serve to direct bacteria to areas with lower amounts of eATP in order to invade the territory and disperse from infected tissue [15]. Moreover, *P*. *aeruginosa* possibly uses the host-derived eATP at sites of epithelial cell damage to detect potential infection sites [15]. It was further argued that eATP acts as a virulence factor regarding its cytotoxic effects.

Since eATP has been associated with pulmonary inflammation and cystic fibrosis [16, 17], Nolan and colleagues [15] also proposed that eATP plays a crucial role in the pathogenesis and chronic infection with *P*. *aeruginosa*. Nevertheless, extracellular ATP was found to be associated with pathogenesis of uropathogenic bacteria such as *E*. *coli*. Bladder function is regulated by both the sympathetic and parasympathetic nervous system pathways; thus, the bacteria-derived neurotransmitters may potentially play major roles in bladder function [5]. ATP is one of many excitatory compounds that pathogenic bacteria can release and induce Ca^2+^ influx and contraction of myofibroblasts, and eATP may act as a virulence factor affecting the urothelium. Interestingly, this study also investigated the effect of subtherapeutic doses of antibiotic on intracellular pathogens that are exposed to lower concentrations of antibiotic during the treatment. Subtherapeutic exposure to ciprofloxacin caused *E*. *coli* to release even higher levels of ATP which has the potential to enhance bladder contractility [5].

### ATP as a biomarker for bacterial susceptibility to antibiotics

Several studies have employed the measurement of ATP with luciferase-luciferin system as a method for antibiotic susceptibility testing (AST). The most frequent approach for detecting bacterial susceptibility or resistance to antibiotics with ATP assays involves bacterial cell lysis and detection of iATP [18, 19, 21, 22]. However, some studies showed that eATP measurements can also give a reliable indication of bacterial susceptibility to antimicrobials [18, 20]. Moreover, the results demonstrated that ATP bioluminescence assay is the most sensitive, selective and rapid method to determine antibiotic susceptibility, suggesting that it could replace the conventional AST methods, especially considering the difficulties related to reagents and instrumentation [18–22].

Rapid and sensitive tests for antibiotic susceptibility are urgently needed due to the emergence of multidrug resistant bacteria such as methicillin resistant *Staphylococcus aureus* (MRSA) that are becoming a serious threat to public health as they can cause severe pathogenic infections such as sepsis, pneumonia and meningitis [23, 24]. These bacteria are part of the group known as the ESKAPE pathogens (*Enterococcus faecium*, *S*. *aureus*, *Klebsiella pneumoniae*, *A*. *baumannii*, *Pseudomonas aeruginosa*, and *Enterobacter* spp.) which are the leading cause of nosocomial infections worldwide [25]. Most of these strains are multidrug resistant with limited treatment options [26].

### Methods for detection of antibiotic susceptibility

Methods for the determination of bacterial susceptibility/resistance to antibiotics are crucial for selecting effective antibiotic therapies as well as reducing the risk of new multidrug resistant bacterial strains. Most of the currently used AST methods are based on bacterial growth inhibition following antibiotic exposure. The three most commonly used AST methods are the broth microdilution method, the disk diffusion test and the Etest gradient diffusion [27]. Using the broth microdilution test, bacterial growth is assessed by turbidity in liquid media containing a drug, while for disk diffusion and Etest, zones of growth inhibition are measured on an agar plate around a disk, or a strip impregnated with an antibiotic. Although currently available methods provide valuable insights into the effective antibiotic type and concentration, they require long incubation times (16 to 48 h), are hampered by limited comparability between different types of tests and often yield only qualitative results [28]. Also, it can be difficult to ensure reproducibility of microbial cultures and a given test can be limited in the number of antibiotics that can be tested [28]. Diffusion methods can be semi-automated, though they are not suitable for the analysis of slow-growing and fastidious bacteria and the results are affected by numerous physical factors [29]. Similarly, dilution methods require maintenance of optimal testing parameters in addition to the requirement for a large volume of reagents and experimental space, tedious dilution steps (macrodilution), the possibility of false positive results due to long incubation times, and risks of cross-contamination [29]. The main limitations of the Etest are inaccuracy and the inconsistent behaviour of certain antibacterial agents, such as Penicillin, Ciprofloxacin, Ofloxacin, and Rifampicin and pH-sensitive coated antibiotics, expensive batch performance, and strip storage [29]. Although measuring eATP during the bacterial cell death and stress has been recognized as a valuable method for determining antibiotic efficacy and assessing development of antibiotic resistance in specific bacterial strains [18, 20], neither luciferase-based eATP assays nor iATP assays involving cell lysis have been widely used in commercial AST assays.

### ATP-based hygiene and cell viability testing

The ATP bioluminescence assay is based on a two step-oxidation of D-luciferin catalysed by the enzyme luciferase in the presence of Mg^2+^, O_2_ and ATP. The reaction results in the emission of light which can be quantified with ultrahigh sensitivity using a luminometer. The amount of light emitted during the reaction has been shown to be directly proportional to the amount of ATP present in the sample [19]. ATP content in turn can be used to estimate the number of viable cells in a sample [1]. As luciferase cannot pass through the cytoplasmic membrane of living microbial cells, ATP hygiene tests are most often performed in an end-point assay format after cell lysis or ATP extraction, thereby quantifying all ATP present in cells. Various cell lysis and ATP extraction agents have been described for bacterial ATP assays, e.g. dodecyltrimethylammonium bromide [30], dimethylsulfoxide [31] and perchloric acid [2]. Online (real time) measurement of eATP in microbial cultures has not yet been described, presumably due to limited stability of the most widely available wild-type luciferases. The half-life of the WT firefly luciferase in assay buffer at room temperature is only 3 h which makes it difficult to use in growth experiments requiring longer time periods of monitoring [32, 33]. In the last two decades several engineered firefly luciferases have been described with strongly improved thermostability in buffer [34–36]. Such optimized enzymes could therefore be suitable for repeated, long-term measurements of eATP in microbial cultures.

### Existing methods for ATP detection in antibiotic susceptibility and hygiene testing

The luciferin-luciferase assay is the most commonly used ATP-detection method in studies of antibiotic susceptibility [18–22, 28, 37] as well as the cleanliness of surfaces and medical equipment in healthcare facilities or food industries [32, 38–40]. Other methods involve ATP-sensing fluorescent proteins such as engineered luciferase probes expressed on the outer side of the plasma membrane [41, 42] or use of fluorescence microscopy for real-time ATP measurement using a two-enzyme system [43]. These methods were developed in order to overcome the limitations of currently available bioluminescence luciferase-luciferin systems which do not allow real-time and *in vivo* measurements [41]. However, the complexity of these methods doesn’t allow for rapid on-site application and they are predominantly used for eukaryotic systems. On the other hand, the method presented in this work might be a preferable choice when simplicity and rapid results are priority such as in the case of antibiotic susceptibility testing and hygiene tests.

### Luminescence-based method for real-time monitoring of eATP

In this paper, we are introducing a real-time eATP monitoring system using a commercially available thermostable luciferase (X-Shining^™^). The enzyme originally derives from the North American firefly *Photuris pennsylvanica* and has been genetically modified for increased heat and storage stability. To the best of our knowledge, the luciferase-luciferin system is the only method allowing real-time monitoring of eATP. Our monitoring system for eATP facilitates sensitive and rapid detection of live microbial cells and allows real-time assessment of the physiological state of microbial cells on surfaces such as agar and in broth culture. Furthermore, the method is useful for rapid assessment of the response of bacterial cells to antibiotics, representing a novel approach to antibiotic susceptibility testing.

## Results

### Stability of luciferases in growth medium

Wild-type *Photinus pyralis* luciferase is known to be rather unstable in assay buffers and is therefore not suited for continuous measurements of ATP bioluminescence in bacterial cultures.

We tested whether the stability of a commercially available thermostable luciferase (X-Shining^™^ luciferase, product code BX174908, Biosynth Carbosynth) in standard growth medium for bacteria is sufficiently high in order to allow real-time monitoring of extracellular ATP in bacterial cultures for up to 24 h at 37°C. There have been other thermostable firefly luciferases developed with equivalent [44], or similar properties [34–36], which could also be suitable for the real-time assays described in this work.

**Fig 1:**
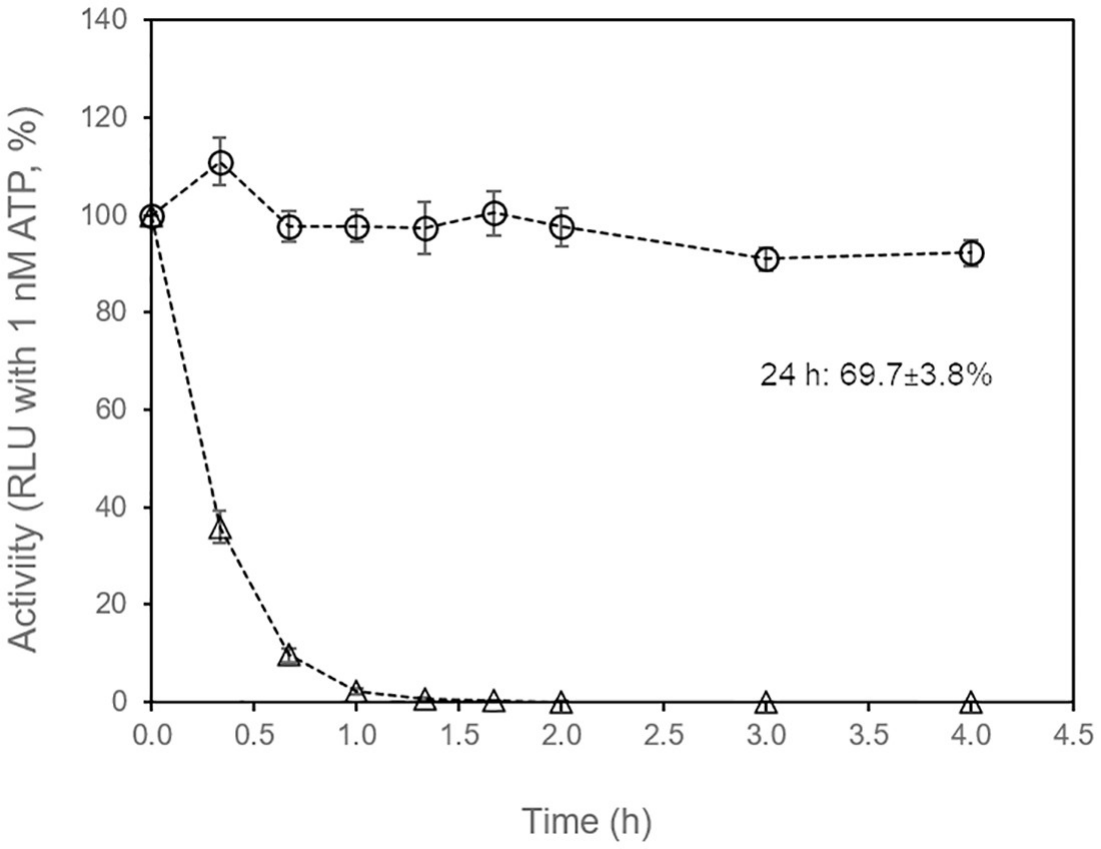
Stability of wild type and thermostable luciferase in medium. Stability of wild-type *Photinus pyralis* luciferase (open triangles) and thermostable luciferase (open circles) was determined by incubating 10 μg/mL enzyme in sterile nutrient broth at 37°C. Luminescence at time zero was set as 100%. Average values of three replicate experiments. Error bars: standard deviation.

Thermostable and wild-type luciferase were added to sterile nutrient broth and enzymatic activity was monitored in regular intervals by withdrawing samples and adding 0.15 mM D-luciferin and 0.4 μM ATP. Engineered, thermostable luciferase retained 90% and 70% of its initial activity in growth medium after 4 h and 24 h at 37°C, respectively (Fig 1). In contrast, conventional, wild-type luciferase lost 80% of its initial activity in growth medium within 30 min and was almost completely inactive after 1 h incubation at 37°C.

The high stability of engineered, thermostable luciferase in medium at optimal incubation temperature prompted us to test whether it could be used for online (real-time) monitoring of extracellular ATP in bacterial cultures as an indicator of bacterial growth and physiological state.

### Luminescence-based monitoring of bacterial growth on agar surface

**Fig 2:**
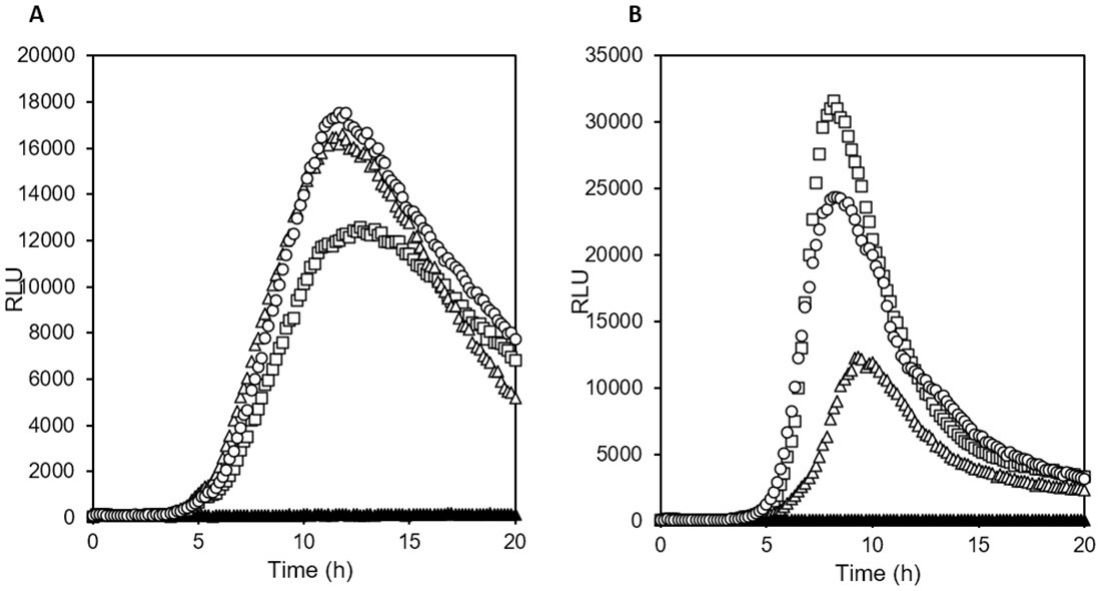
Online (real time) monitoring of eATP released by bacteria growing on agar in a 96-well plate. D-luciferin and thermostable luciferase had been added to molten nutrient agar before solidification. (Open symbols: three replicate wells, agar surface inoculated with 2 μL bacterial cell suspension; closed symbols: sterile control well). (A) *Staphylococcus aureus* ATCC 25923, 2.5*10^4^ CFU inoculated per well, (B) agar *Escherichia coli* ATCC 25922, 3.4*10^3^ CFU inoculated per well. Incubation temperature was 37°C.

**Fig 3:**
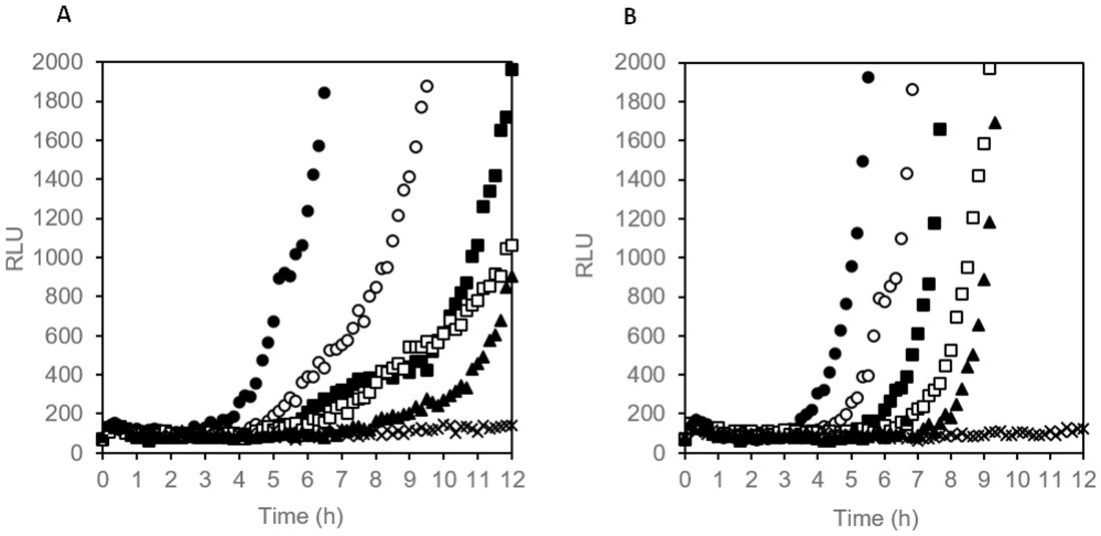
Onset of eATP accumulation in agar cultures in relation to inoculation density. Agar in a 96-well plate contained 10 μg/mL thermostable luciferase and 0.15 mM D-luciferin. (A) *S*. *aureus*, curves with alternating filled and closed symbols from left to right, per well: 2.5*10^4^ CFU, 2.5*10^3^ CFU, 2.5 10^2^ CFU, 25 CFU, 3 CFU, crosses: sterile control. (B) *E*. *coli*, curves with alternating filled and closed symbols from left to right, per well: 4.6*10^3^ CFU, 4.6*10^2^ CFU, 46 CFU, 5 CFU, approx. 1 CFU; crosses: sterile control.

First, we examined whether it is possible to follow eATP released by bacteria growing on agar surfaces. Wild-type luciferase and thermostable luciferase were added to molten nutrient agar together with D-luciferin. Solidified agar in wells of a white 96-well plate was inoculated with small drops of cell suspension on the centre of the surface. When luminescence was measured through a transparent lid from the top, growth of *E*. *coli* and *S*. *aureus* on agar containing thermostable luciferase and D-luciferin resulted in reproducible patterns of luminescence increase and subsequent decrease (Fig 2). Both *S*. *aureus* (2A) and *E*. *coli* (2B) showed an exponential increase in luminescence at the beginning, followed by a linear increase and then a plateau or peak. Peak luminescence intensity, but not peak time varied between individual cultures of the same strain, presumably due to the complexities of colony growth. As expected, no increase of luminescence was observed in sterile control wells. The onset time of detectable luminescence increase was proportional to the number of cells inoculated on the agar surface (Fig 3). Inoculation with single digit numbers of cells could be detected after culturing for 8 to 10 h, which is considerably faster than incubation time required for visual detection of colonies on agar plates (typically 16 to 20 h).

### Real-time monitoring of bacterial growth in broth culture via eATP

**Fig 4:**
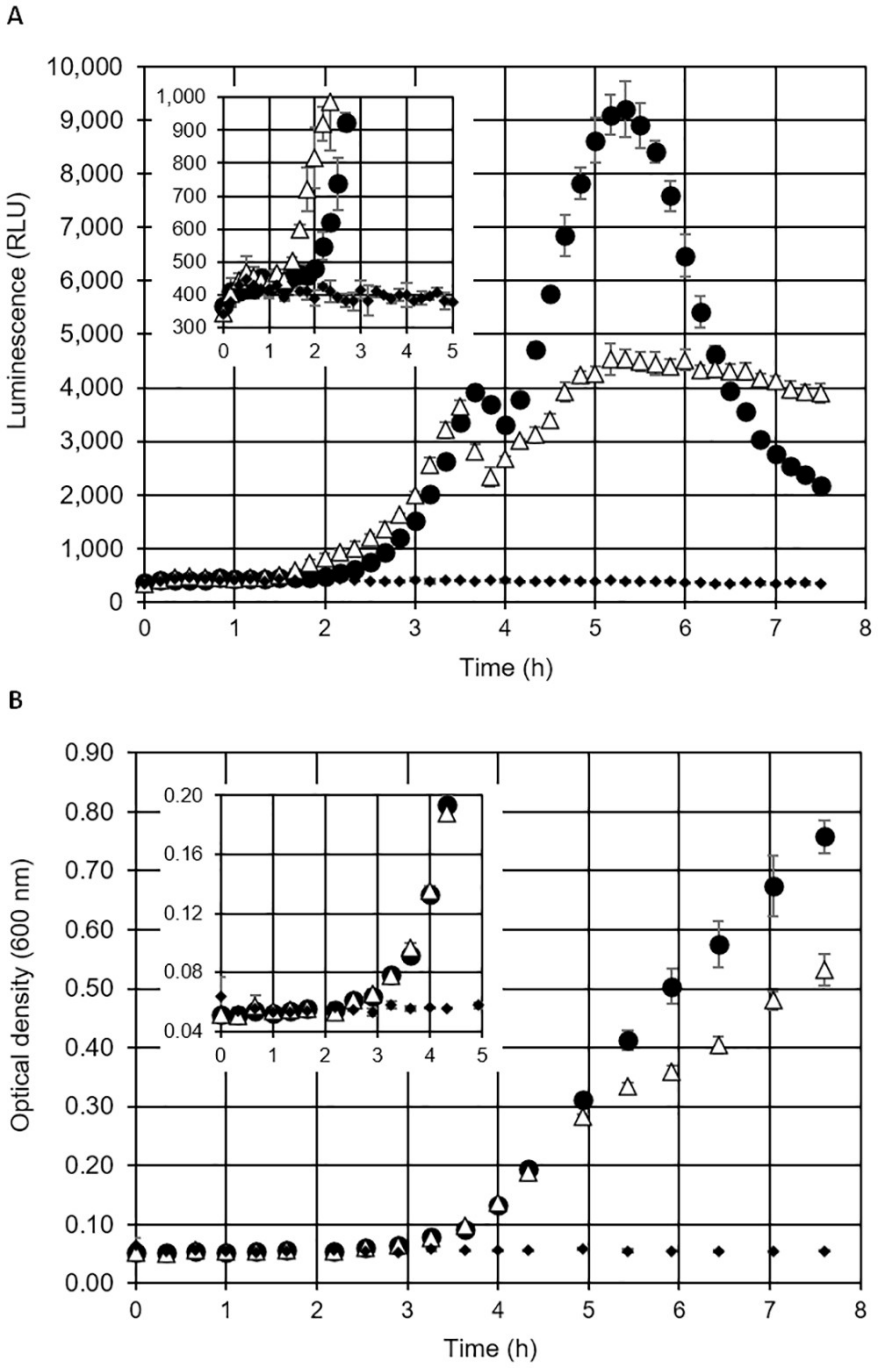
Monitoring of bacterial growth in nutrient broth by eATP detection and optical density. The bacterial growth in liquid media was monitored by (A) online measurement of luminescence (eATP, with thermostable luciferase and D-luciferin) and (B) measurement of optical density. Filled circles: *S*. *aureus*, open triangles: *E*. *coli*, filled diamonds: sterile control. Starting cell density was 10^5^ CFU/mL. Average values of three replicate experiments, error bars represent standard deviation.

Analysis of cell multiplication in broth culture is indispensable for the study of microbial physiology and is normally done by measurement of turbidity (optical density). We tested if bacterial growth in nutrient broth can also be measured in real-time by adding thermostable luciferase and D-luciferin to the medium, facilitating detection of eATP accumulating over time. *Escherichia coli* (Gram negative) and *Stapyhlococcus aureus* (Gram positive) showed distinct, but reproducible patterns of luminescence, with exponential increase at the beginning followed by a plateau in the case of *E*. *coli*, and a peak followed by a decline in the case of *S*. *aureus* (Fig 4A). Detection of growth at 37°C, when starting from 10^5^ CFU/mL, was possible after 2 to 2.5 h with the online luciferase method. For comparison, growth at 37°C was also analysed by turbidity measurements (OD_600_) in experiments with the same starting cell concentration and the same medium. An increase in optical density, sufficient to distinguish inoculated broth from sterile control was observed 1 to 2 h later (Fig 4B) than an increase in eATP luminescence, indicating that the luminescence method for monitoring growth is approximately 10-fold more sensitive (assuming a doubling time of 20 min). From both luminescence and optical density (OD_600_) data it in can be concluded that fast accumulation of extracellular ATP occurs mainly in the phase of unrestricted, exponential growth at low cell density (OD_600_ < 0.1.). In microwells it is likely that cultures adapt to oxygen limitation already at rather low OD, contributing to a slowdown in eATP accumulation after switching to less efficient anaerobic catabolism.

### Ratio of eATP relative to total ATP

In order to quantify the amount of eATP relative to total ATP we carried out endpoint measurements in 96-well plates after 3 and 5 h incubation time at 37°C, using the same inoculation density of *E*. *coli* and *S*. *aureus* as in Fig 4. Luminescence (relative light units, RLU) was measured immediately before and after addition of the cell lysis reagent dodecyltrimethylammonium bromide (DTAB). ATP concentrations in nanomole per liter (nM) were calculated from net RLU values (background RLU of sterile control subtracted) using an RLU/nM ATP coefficient derived from an ATP standard curve in nutrient broth with thermostable luciferase and D-luciferin. Luminescence showed a linear correlation with added ATP up to a concentration of 50 nM (S1 Fig). The concentration of eATP and total ATP in microwell cultures ranged from 0.2 to 5.4 nM and 8.5 to 32.5 nM, respectively, depending on strain and incubation time (Table 1). The relative amount of eATP was 3 to 5% of total ATP in the early growth phase after 3 h and increased to 11–17% after five hours of growth at 37°C (Table 1). Background luminescence from medium ATP in sterile controls after 5 h incubation at 37°C was 529±36 (average and standard deviation of n = 3 replicate wells). Assuming that three times the standard deviation of the average background value represents the detection limit of eATP in nutrient broth, the minimal concentration of eATP detectable in microwell cultures is approximately 0.03 nM ATP.

**Table 1:** eATP and total ATP concentrations in cultures of *S*. *aureus* and *E*. *coli* in nutrient broth containing thermostable luciferase and D-luciferin.

	3 h, 37°C		5 h, 37°C	
	ATP [nM]	% eATP	ATP [nM]	% eATP
*S*. *aureus*, eATP	0.242 (±0.048)	2.8	5.35 (±0.54)	16.5
*S*. *aureus*, total ATP (DTAB lysis)	8.68 (±4.13)		32.4 (±4.7)	
*E*. *coli*, eATP	0.390 (±0.057)	4.6	1.48 (±0.04)	10.8
*E*. *coli*, total ATP (DTAB lysis)	8.46 (±2.62)		13.8 (±0.69)	

Starting cell density was 10^5^ CFU/mL, incubation temperature was 37°C. Average values of three replicate cultures in 96-well plate, standard deviation is given in parentheses.

### Estimation of number of viable cells by luminescence onset time in liquid culture

**Fig 5:**
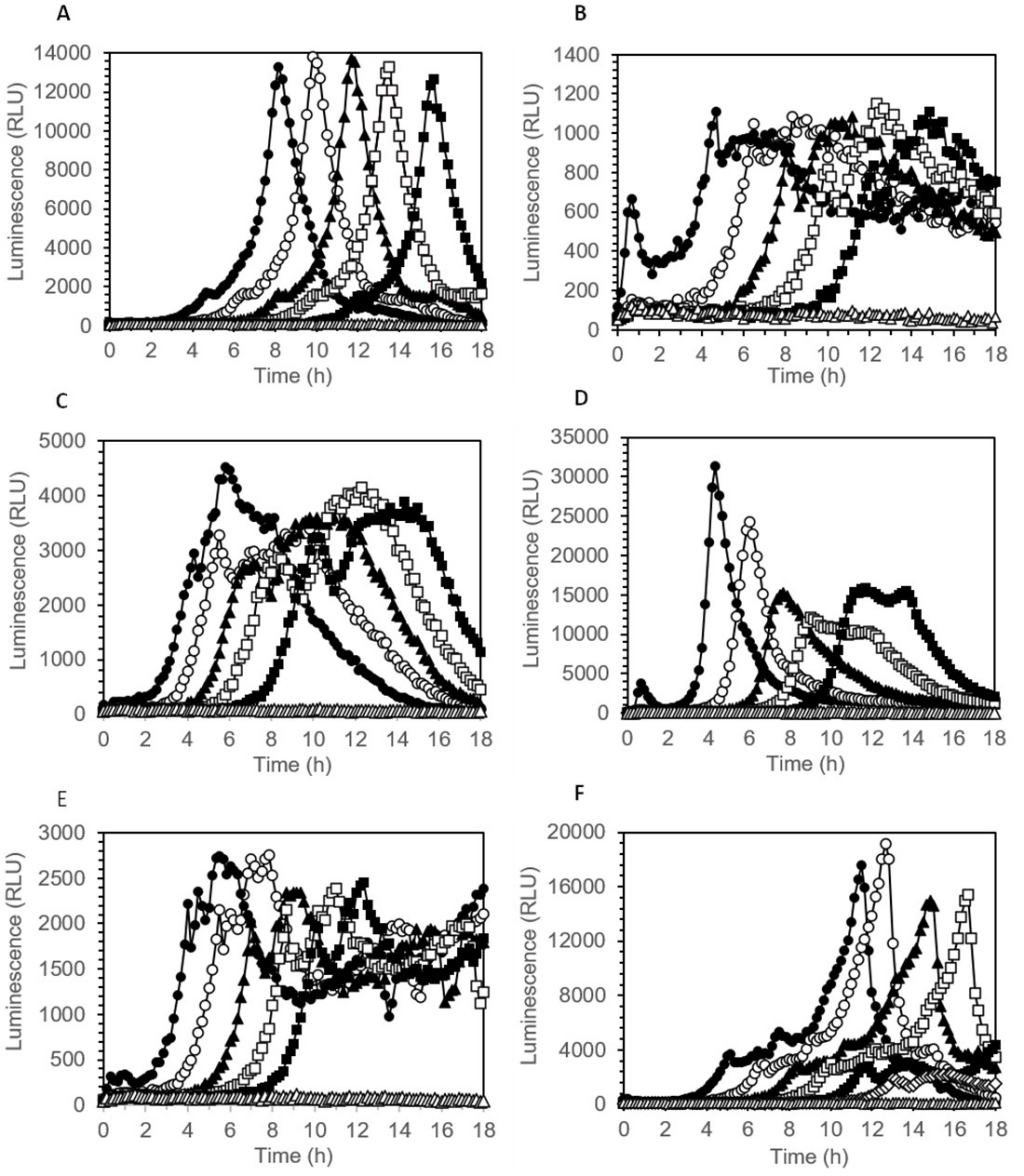
eATP pattern of bacterial broth cultures measured online in medium containing thermostable luciferase and D-luciferin. A 96-well plate was incubated at 30°C. Initial cell concentrations were as follows: 10^6^ CFU/mL (closed circles), 10^5^ CFU/mL (open circles), 10^4^ CFU/mL (closed triangles), 10^3^ CFU/mL (open squares), 10^2^ CFU/mL (closed squares), 0 (sterile control, open triangles). (A) *Pseudomonas fluorescens*, (B) *Pantoae agglomerans*, (C) *Bacillus cereus*, (D) *Staphylococcus aureus*, (E) *Escherichia coli*, (F) mixture of normalised cell suspensions of all five bacterial species.

**Fig 6:**
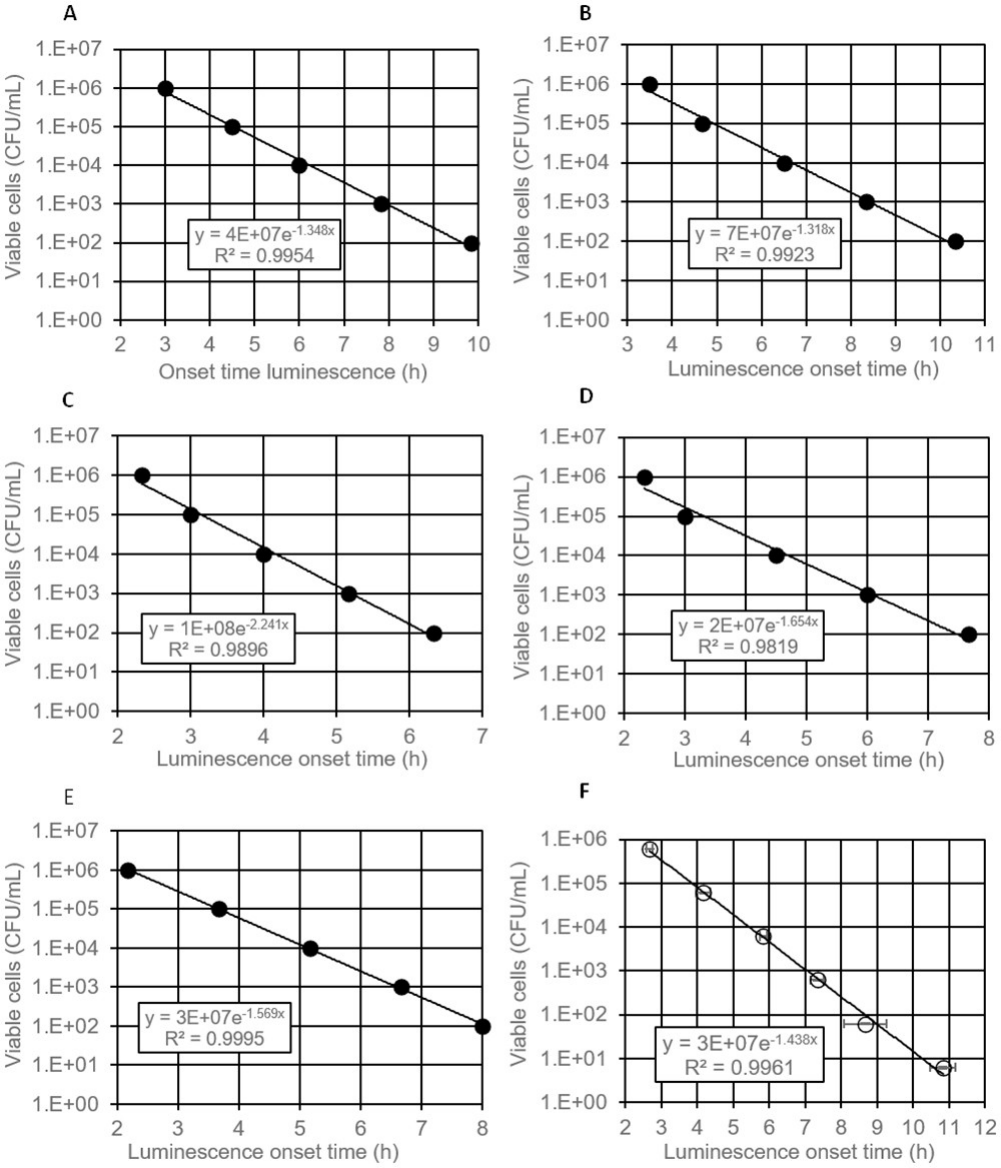
Correlation of luminescence onset time with initial concentration of viable cells. 96-well plate was incubated at 30°C. (A) *Pseudomonas fluorescens*, (B) *Pantoae agglomerans*, (C) *Bacillus cereus*, (D) *Staphylococcus aureus*, (E) *Escherichia coli*, F mixture of normalised mixtures of all five bacterial species (A-E: single wells, F: average values of three replicate wells, error bars represent standard deviation).

The conventional method for determining the number of viable bacterial cells in a sample is plating on a complex medium agar, usually requiring incubation times of 16 h to up to 48 h to obtain visible colonies. We investigated whether online (real-time) measurement of eATP accumulation in broth culture is suitable for estimation of the concentration of viable cells initially present in samples. We hypothesized that the time required for accumulation of detectable levels of eATP during rapid, exponential growth (designated “luminescence onset time”) correlates with the initial cell concentration. All five tested bacterial species from widely differing phylogenetic families showed eATP accumulation in broth culture (Fig 5). The eATP increase was exponential at the beginning, later levelled off and then decreased again or remained constant, depending on the strain. Interestingly, the temporal pattern of eATP accumulation and decline varied strongly between different species, but was highly reproducible in replicate cultures of the same species (Fig 5), even at strongly varying initial cell densities. As predicted, the onset time of a significant luminescence increase occurred the later the lower the inoculation density had been (Fig 5). We defined a minimum slope of 150 RLU/h (relative light units per hour) of four subsequent time points as threshold for designating an onset time in a microwell culture. When plotted against the initial cell concentration in log scale, a negative linear correlation of initial cell density with luminescence onset time was obtained, facilitating quantification of viable cell concentration (Fig 6). The slope of the regression line varied between different species, reflecting differences in specific growth rates. A strong correlation between initial cell concentration and luminescence onset time was also observed for mixtures of all five species (Fig 6E), indicating that the method can also be used for estimation of viable cell counts in settings where a diverse community of bacteria is present.

### Effect of antimicrobial agents analysed with eATP online assay

We tested whether the effect of antimicrobial agents can be monitored online (in real-time) by following eATP dynamics in medium containing thermostable luciferase and D-luciferin. Beta-lactam antibiotics interfere with the synthesis of the bacterial cell wall and are one of the most important classes of compounds used for treatment of bacterial infections in medicine. We chose Ampicillin as an example of a penicillin derivative and Imipenem as an example of a carbapenem. Carbapenems have been developed for treatment of bacteria resistant to other beta-lactam antibiotics. In addition, we tested the effect of Polymyxin B, an agent that disrupts membrane integrity. We compared the effect of these three antimicrobial agents on a standard, antibiotic sensitive strain of *E*. *coli* and an *E*. *coli* strain harbouring the *ampC* gene encoding a potent β-lactamase, conferring resistance to numerous cephalosporins and most penicillins [30]. The strains were inoculated at approx. 10^5^ CFU/mL to nutrient broth containing thermostable luciferase and luciferin in a 96-well plate. The plate was incubated for 1 h at 37°C, then either Imipenem, Ampicillin or Polymyxin B were added from 100-fold concentrated stock solution and incubation continued (perturbation assay). Control cultures were left undisturbed. Luminescence was measured throughout the experiment.

**Fig 7:**
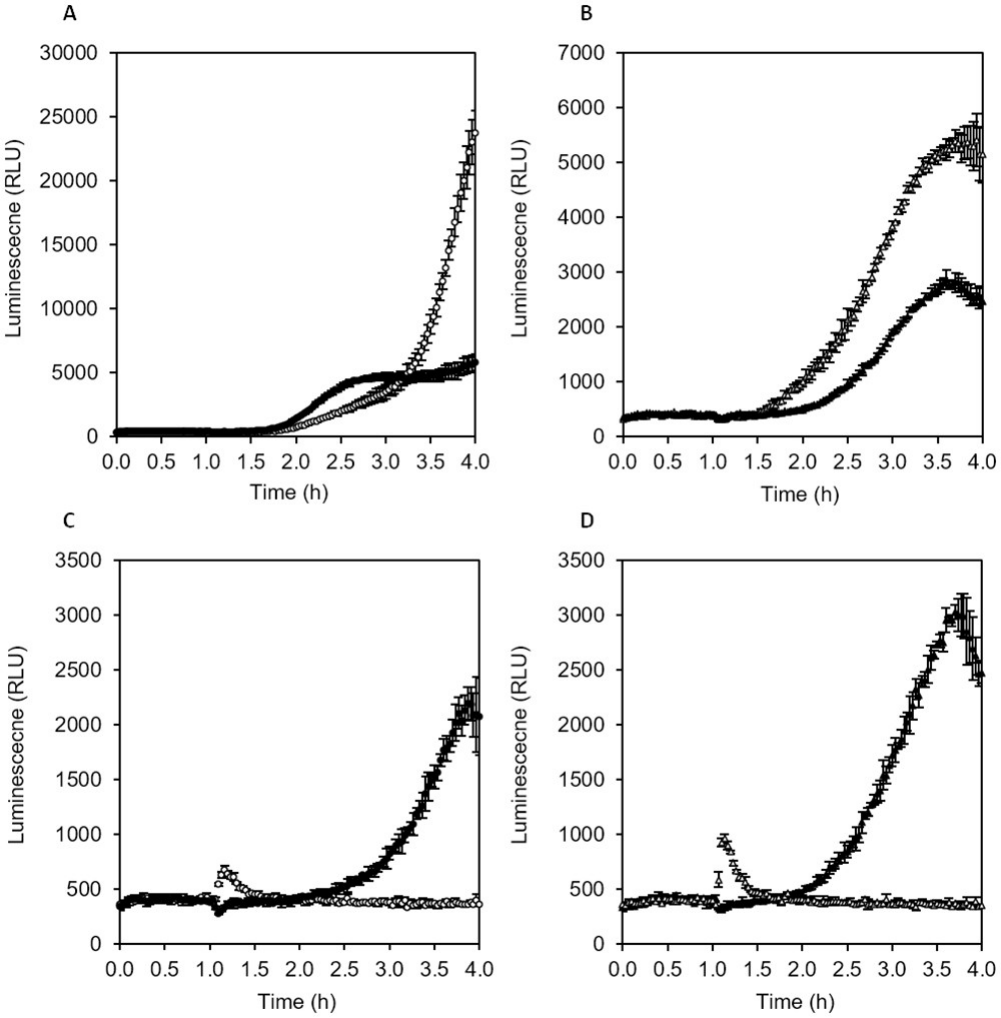
Effect of antimicrobial agents on eATP pattern in bacterial cultures. Nutrient broth containing thermostable luciferase and D-luciferin in a 96-well plate was inoculated with 10^5^ CFU/ml *E*. *coli* and incubated at 37°C in a plate reader. Antimicrobial agents were added after 1 h. (A) Antibiotic sensitive *E*. *coli* ATCC25922 with 50 μg/mL Ampicillin (filled circles) or 4 μg/mL Imipenem (open circles), (B) antibiotic resistant *E*. *coli* RKI 66/09 AmpC (CMY-2) with 50 μg/mL Ampicillin (filled triangles), or 4 μg/mL Imipenem (open triangles), (C) Sensitive *E*. *coli* without antibiotics (filled circles) or with 50 μg/mL Polymyxin B (open circles), (D) resistant *E*. *coli* without antibiotics (filled triangles), or with 50 μg/mL Polymyxin B (open triangles).

Ampicillin as well as Imipenem strongly increased leakage of ATP from actively growing antibiotic sensitive *E*. *coli* ATCC25922 within 1 to 1.5 h of exposure (Fig 7A, compared to control without antibiotic in Fig 7C). By contrast, Ampicillin did not accelerate and increase the amount of eATP in cultures of antibiotic resistant *E*. *coli* RKI 66/09 AmpC (CMY-2) (Fig 7B, compared to control without antibiotic in Fig 7D). Imipenem increased leakage of ATP also in the resistant strain, although not as strongly as in the sensitive strain, indicating that this antibiotic is effective for both strains. Addition of Polymyxin B caused immediate release of eATP in cultures of both strains (Fig 7C and 7D). The luminescence peak disappeared again quickly, and luminescence returned back to baseline level, indicating that generation of ATP had ceased and cells were dead. Polymyxin B-treated cultures were clearly distinguishable from control cultures without antimicrobial agents where growth-related accumulation of eATP was obvious after 2.5 to 3 h. The results show that it is possible to distinguish between bacterial strains which are sensitive or resistant to antibiotics based on their eATP profile.

The luminescence pattern in replicate cultures with the same antibiotic were highly reproducible, therefore analysis of the effect of known and novel antimicrobial agents on microbial cells using online (real-time) eATP luminescence assays seems to be a valid approach.

Next, we tested whether the online eATP method can also be used directly with resting cells and if it is suitable for determination of the minimal inhibitory concentration (MIC) of antibiotics. Decreasing concentrations of Ampicillin were added to nutrient broth containing thermostable luciferase and D-luciferin in a white 96-well plate. Wells were inoculated with approx. 2·10^6^ CFU/mL of antibiotic sensitive or antibiotic resistant *E*. *coli* (same strains as above), which had been grown overnight at 37°C and 150 rpm in nutrient broth (stationary phase cells). The plate was incubated at 37°C in a plate reader and luminescence was followed for 4 h.

**Fig 8:**
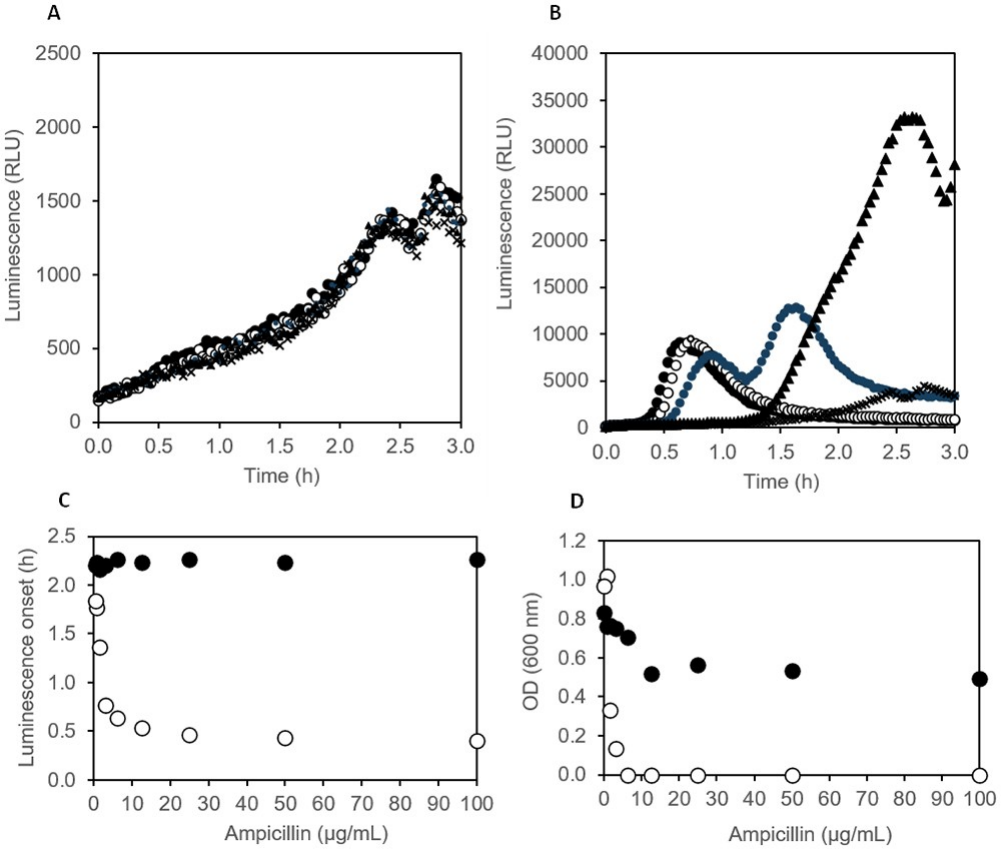
Effect of different concentrations of Ampicillin on eATP release pattern in antibiotic resistant *E*. *coli* RKI 66/09 AmpC (CMY-2) and antibiotic sensitive *Escherichia coli* ATCC 25922. (A) Antibiotic resistant *E*. *coli* with 100 μg/mL (filled circles), 25 μg/mL (open circles), 6.25 μg/mL (shaded circles), 1.56 μg/mL (filled triangles) and 0 μg/mL (crosses) Ampicillin; (B) Antibiotic sensitive *E*. *coli* with 100 μg/mL (filled circles), 25 μg/mL (open circles), 6.25 μg/mL (shaded circles), 1.56 μg/mL (filled triangles) and 0 μg/mL (crosses) Ampicillin; (C) luminescence onset time (first time point with RLU above 1200) in dependency of Ampicillin concentration for antibiotic resistant (closed circles) and antibiotic sensitive *E*. *coli* (open circles); (D) optical density after 15 h incubation in microplate wells in dependency of Ampicillin concentration for antibiotic resistant (closed circles) and antibiotic sensitive *E*. *coli* (open circles). Inoculation density was 2·10^6^ CFU/mL in all experiments, cells were taken from stationary phase cultures in nutrient broth.

Optical density in well cultures was measured after overnight incubation to check how well the results of the classical broth dilution technique can be reproduced with the luciferase online method. In the case of the Ampicillin-resistant *E*. *coli*, neither the pattern of eATP accumulation nor growth as measured by OD_600_ was affected by Ampicillin up to the highest tested concentration of 100 μg/mL, indicating that the minimal inhibitory concentration had not been reached (Fig 8A, 8C and 8D). Luminescence remained low for the first hour and never surpassed values of 2000 RLU until the end of the experiment. In contrast, the Ampicillin-sensitive *E*. *coli* released high amounts of ATP within 45 min at Ampicillin concentrations of 6 μg/mL and higher (Fig 8B, 8C and 8D). Similar to the resistant strain, luminescence in the control culture of the sensitive strain without antibiotic remained low for 1.5 h and then continuously increased until around 4000 RLU was reached after 3 h. At an Ampicillin concentration of 1.6 μg/mL, which only partially inhibited growth, the sensitive strains leaked massive amounts of ATP as judged by the luminescence pattern (Fig 8B), indicating that cell wall synthesis was disturbed, but metabolism and biosynthesis remained partially functional. When defining 1200 RLU as a threshold for luminescence onset, the time when the threshold was surpassed could be plotted against the Ampicillin concentration (Fig 8C). The shape of the luminescence onset time curves of both the sensitive and the resistant strains matched the shape of the Ampicillin concentration–OD_600_ curves (Fig 8D). As can be seen, the luminescence onset time inversely correlated with Ampicillin concentration for antibiotic sensitive *E*. *coli*, whereas essentially the same onset time was observed at all tested Ampicillin concentrations in the case of antibiotic resistant *E*. *coli*. A minimal inhibitory concentration of approximately 6 μg/mL could be deduced from the luminescence onset time curve of antibiotic sensitive *E*. *coli* (Fig 8C), which was consistent with the minimal inhibitory concentration deduced from OD_600_ measurements (Fig 8D).

**Fig 9:**
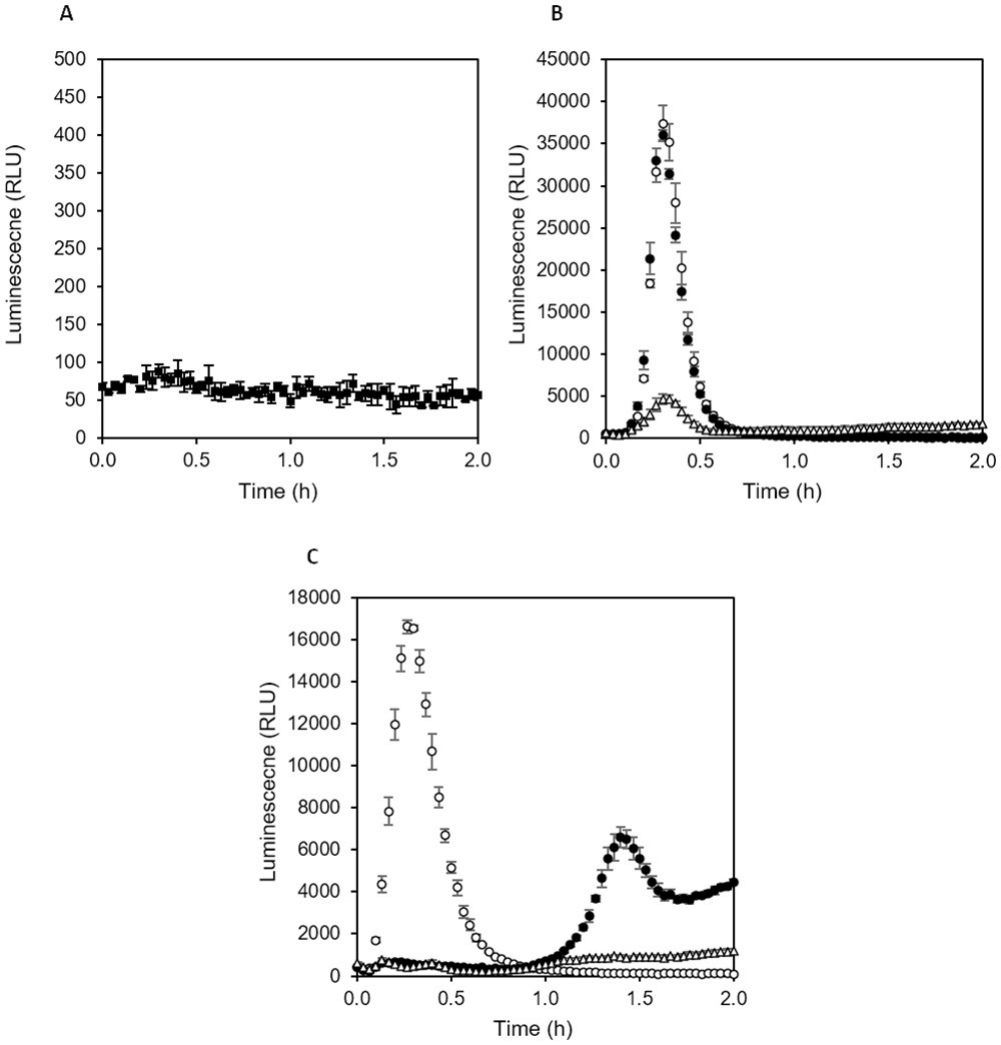
eATP release pattern in presence and absence of antibiotics in low strength medium with glucose and pyruvic acid. (A) Sterile medium, (B) *E*. *coli* ATCC25922 (antibiotic sensitive) with 50 μg/mL Ampicillin (filled circles), 50 μg/mL Imipenem (open circles) and without antibiotics (open triangles), (C) *E*. *coli* RKI 66/09 AmpC (CMY-2) (antibiotic resistant) with 50 μg/mL Ampicillin (filled circles), 50 μg/mL Imipenem (open circles) and without antibiotics (open triangles). The medium contained thermostable luciferase and D-luciferin. Inoculation density was 10^6^ CFU/mL, cells were taken from stationary phase cultures in nutrient broth, incubation temperature in plate reader was 37°C. Average values of three replicate cultures in 96-well plate, error bars represent standard deviations.

Finally, we tested whether the online eATP method can be further optimized by switching to different media. The standard complex medium Nutrient broth contains 2 g/L yeast extract and 1 g/L meat extract, both contributing to ATP background in luminescence assays. Background in nutrient broth supplemented with luciferin and thermostable luciferase remained in the range of 400 to 600 RLU in our system, even after prolonged pre-incubation at 37°C for 3 to 4 hours. Media with reduced content of complex components may facilitate lower background and thus a higher sensitivity of online eATP assays. Furthermore, addition of easily metabolizable carbon- and energy sources such as glucose and pyruvic acid may lead to accelerated and increased release of eATP. In fact, a medium containing approximately 10-fold lower concentrations of complex components (0.1 g/L yeast extract, 0.05 g/L peptone, 0.05 g/L tryptone) as wells as 0.5 g/L glucose, 0.5 g/L glycerol and 0.1 g/L sodium pyruvate showed a 10-fold reduced background luminescence of 60 to 70 RLU after burn-off (Fig 9A). Furthermore, eATP released in the presence of beta-lactam antibiotics when wells were inoculated with stationary phase cells peaked at 17000 to 37000 RLU after just 20 min (Fig 9B and 9C), which is substantially stronger and faster compared to 10000 RLU reached after 40 min in nutrient broth (Fig 8B). In agreement with results obtained in nutrient broth, the antibiotic resistant strain showed no eATP release in the presence of Ampicillin within the first hour, while Imipenem caused a strong and fast eATP peak (Fig 9C). With the optimized medium, a signal-to-background ratio of up to 600 could be reached. Interestingly, the sensitive strain showed a significant eATP release after 20 min even without antibiotics, while the resistant strain showed no such response (Fig 9B and 9C).

The online eATP assays shown in Figs 7 to 9 lead us to the conclusion that *E*. *coli* ATCC 25922 was inhibited and/or killed by all three antimicrobial agents, while *E*. *coli* RKI 66/09 AmpC (CMY-2) was only inhibited/killed by Imipenem and Polymyxin B, but not by Ampicillin. We were able to confirm this conclusion in tube cultures with the low strength medium supplemented with either 4 μg/mL Imipenem, 50 μg/mL Ampicillin, 50 μg/mL Polymyxin B or without antimicrobial agent. Optical density (600 nm) after overnight incubation at 37° C showed growth of *E*. *coli* ATCC25922 only in the control culture without antimicrobial agents, while growth of *E*. *coli* RKI 66/09 AmpC (CMY-2) was inhibited by Imipenem and Polymyxin B, but not by Ampicillin (S2 Fig).

## Discussion

Luciferase based measurements of ATP in microbiology was until now mostly done using endpoint assays with cells lysis, measuring either total ATP or intracellular ATP when an ATP degrading enzyme such as apyrase is added before lysis. Few studies have reported the measurement of eATP in endpoint assays [2] and although eATP may play an important role in bacterial physiology and pathogenicity it is not well studied. A novel and convenient way to monitor eATP over time would help elucidate its roles in various processes, not only in bacterial but also in human physiology and pathophysiology, including host-microbiome interactions. Also, online eATP bioluminescence assays may have great potential for assessing of bacterial cell viability and susceptibility to antibiotics.

In the present study, we used a luciferase-luciferin system with a genetically engineered, thermostable luciferase (X-Shining^™^) that enabled real-time monitoring of extracellular ATP in bacterial cultures. The thermostable luciferase was shown to be suitable for assays in liquid and on solid media, giving opportunity to study eATP dynamics not only with a luminometer, but also using different techniques, such as time-lapse microscopy experiments, where bacteria are grown on small agar pads [33]. We showed that the peak of eATP release from *E*. *coli* and *S*. *aureus* cells occurs in the early log phase by measuring bioluminescence which is consistent with previous findings of Mempin and colleagues [2]. eATP concentrations measured in our experiments were in the same order of magnitude as in the study of Mempin *et al*. [2] who reported 3 to 18 nM eATP in culture supernatants of *E*. *coli* and *Salmonella*, together with an eATP to intracellular ATP ratio of 0.1 and 0.6 for two different strains of *Acetinobacte*r; the latter being a strain with extraordinarily high levels of eATP. With 3 to 16% eATP relative to total ATP observed in our experiments with *E*. *coli* and *S*. *aureus*, our results are in the range of the low eATP strain of Mempin *et al*. [2]. An interesting observation in our experiments was that different bacterial species showed widely varying temporal patterns and peak values of eATP bioluminescence. eATP patterns in replicate cultures of the same strain, however, were well reproducible. Differences in peak eATP levels between different bacterial species were also reported by Mempin *et al*. [2]. We assume that eATP levels in bacterial cultures are determined by the interplay of accidental leakage during cell division, accidental or induced cell lysis, active ATP secretion and active ATP uptake. All of these processes may differ between different species and even between different strains of the same species in response to the growth environment. It is likely that growth conditions in wells of 96-well plates with cover switch from oxic to anoxic/microaerophilic during the exponential growth phase, which is likely to influence ATP levels. When cell growth ends due to nutrient and/or oxygen limitation (stationary phase), both extra- and intracellular ATP levels are expected to drop, which is in agreement with eATP patterns observed for *P*. *fluorescens*, *B*. *cereus* and *S*. *aureus* (Fig 5), all of which are obligate aerobic bacteria. By contrast, eATP levels remained at a constant, relatively low level for extended periods of time in the case of the facultative anaerobic bacteria *E*. *coli* and *P*. *agglomerans* (Fig 5), which could be due to a switch to slow fermentative growth.

The fact that the majority of ATP remains inside bacterial cells leads us to the conclusion that the online eATP assay is less sensitive than endpoint bioluminescence viability tests with cell lysis measuring iATP [31]. However, monitoring of growth with the online eATP bioluminescence assay is 10-fold more sensitive than analysis of growth by repeated measurement of turbidity/optical density. Another advantage of our assay is that all components are added at the beginning of the experiment and no further manipulation is needed. We examined whether real-time measurement of eATP accumulation in broth culture is suitable for estimation of the concentration of viable cells initially present in samples. The onset time of detectable luminescence increase was dependent on inoculation density which enabled an accurate assessment of the number of viable cells, both for Gram positive and Gram negative bacteria and for a mixture of different species. A similar quantitative proliferation assay for analysis of the number of bacteria surviving on different surfaces has been described where growth is measured online in 96-well plates via optical density [45, 46]. Our assay is expected to be significantly faster and could also be performed directly on opaque surfaces, making transfer of samples to a second proliferation assay plate unnecessary. Furthermore, the online bioluminescence assay can most likely also be applied to aggregate-forming and filamentous microorganisms as well as biofilms. Of particular interest is the capability of our assay to monitor solid state cultures on agar. Except for laborious and error-prone quantification of colony size by microscopy, there are few options to monitor growth in such settings.

ATP leakage detection methods have been found to be valuable in identifying an effective antibiotic and determining antibiotic resistance in specific bacterial strains. ATP bioluminescence was previously used to assess antibiotic susceptibility, where gentamicin dose-dependent effects were detected based on eATP in bacterial cultures [18]. Similarly, an ATP bioluminescence assay was used to determine bacteriocin threshold against a target sensitive strain [20]. However, most e diagnostic laboratories still use conventional AST methods, despite ATP bioluminescent assays showing similar or greater accuracy and requiring a shorter time-to-result [18, 20]. A recent study by Heller and Spence [28] described a rapid antibiotic susceptibility test by measuring the ATP/OD_600_ ratio, which provided more quantitative results compared to conventional AST methods. Previously described ATP bioluminescence assays for AST testing rely on a multi-step procedure involving cell lysis and requires 3–4 h until results are obtained. The method presented here based on real-time measurement of eATP is a one-step procedure yielding results within 30 min to max. 2 h. Bioluminescence AST assays have potential to solve several limitations of the conventional AST methods. For example, the most frequently used AST methods, disk diffusion, Etest and broth dilution are time-consuming since bacteria from a patient sample must initially be grown to a suitable cell density for testing before being exposed to different antibiotics in growth assays which again require a substantial incubation time. Therefore, results are only available three days after the sample is taken at the earliest. In contrast, the method described here provides much faster results. Moreover, we showed that MIC can be deduced using the proposed method, as demonstrated with Ampicilin sensitive *E*. *coli*. In addition, subinhibitory doses of antibiotic caused sensitive *E*. *coli* to release high amounts of ATP, which is consistent with previous reports of Abbasian and colleagues [5]. Furthermore, the efficacy of an antibiotic can be rapidly determined within less than 1.5 h and at cell concentrations as low as 10^5^ CFU/mL, as demonstrated with resistant and sensitive *E*. *coli* strains, challenged with Ampicilin, Imipenem and Polymyxin B. Hence, this assay has the potential to be routinely used in clinical diagnostics and to aid in decision making when prescribing antibiotics to patients. Reduced time-to-result is critically important since it allows more rapid administration of narrow-spectrum antibiotics in a clinical setting, leading to improved patient outcomes, lower costs, as well as a lower risk of both, antibiotic side effects and the development of antibiotic resistance.

In conclusion, the ATP bioluminescence assay has a wide-ranging usefulness across various research fields and application areas. Improving the efficiency of this assay opens numerous possibilities in revealing the role of eATP in the physiology of different organisms, as well as in infection and disease control. It is also worth mentioning that the ATP assays have been increasingly employed as a rapid, on-site real-time detection method in nosocomial infection control [35], including screening of household surfaces, hands, and medical equipment [1]. We believe that the above described method for the real-time measurement of eATP will open the door to new discoveries and applications in research and diagnostic settings. In terms of diagnostics, we showed the potential and advantages of the method for the determination of bacterial resistance and susceptibility to antibiotics with Gram positive and negative model bacteria. As the imminent threat of antibiotic-resistant bacteria is rising as is a global concern in the infectious disease field, developing rapid and reliable diagnostic tools is of the utmost importance. Although the present study demonstrated the utility of the method in a microbiological setting, it is important to highlight that it is not limited to the use in prokaryotic organisms. Aside from roles in bacterial physiology, numerous studies reported different roles of eATP in eukaryotic organisms including human and plant physiology [17, 39, 47]. As a universal molecule of all living beings, eATP plays important roles in bacterial, animal and plant kingdoms, thus we believe that this method will allow elucidating the role and dynamics of eATP in different organisms.

## Materials and methods

### Chemicals and enzymes

Wild-type *Photinus pyralis* luciferase (QuantiLum, E1701) was obtained from Promega. Engineered, thermostable luciferase (X-Shining^™^ luciferase, BX174908) and D-Luciferin, potassium salt (FL08608) were obtained from BiosynthCarbosynth. Other chemicals and growth medium components were obtained from Merck in research-grade quality if not indicated otherwise.

### Stability of luciferases in medium

Stability of wild-type *Photinus pyralis* luciferase and engineered, thermostable luciferase was tested in a standard complex growth medium (nutrient broth, 5 g/L peptone, 5 g/L sodium chloride, 2 g/L yeast extract, 1 g/L meat extract, pH 7.4, autoclaved). Enzymes were added at 10 μg/mL final concentration. Medium with luciferase was filter-sterilized and incubated in glass tubes at 37°C and 50 rpm. Luciferase activity was analysed in regular intervals in an opaque, white 96-well plate. Assays were carried out as follows: 0.19 mL medium sample with luciferase was added per well and equilibrated to room temperature for two minutes. Then, 10 μL 20x adenosin-3-phosphate (ATP)/D-luciferin stock solution (prepared with ultrapure water) was added with a multichannel pipet and luminescence (relative light units, RLU) was measured immediately from top every 10 seconds for 3 minutes using a SpectraMax M5 plate reader (Molecular Devices). Final concentration of ATP and D-luciferin were 0.4 μM and 3 mM, respectively. Experiments were performed in triplicate and relative average total luminescence in 3 min (% RLU) was calculated with RLU value of first time point of each experiment set as 100%.

### Online monitoring of eATP in agar cultures

Extracellular ATP (eATP) released during growth of bacteria on agar surface (solid state culture) was analysed by adding either wild-type *Photinus pyralis* luciferase or engineered, thermostable luciferase to molten nutrient agar (5 g/L peptone, 5 g/L sodium chloride, 2 g/L yeast extract, 1 g/L meat extract, 15 g/L agar, pH 7.4) after autoclaving and cooling to 50°C. Thermostable luciferase (10 μg/mL final concentration), 0.15 mM D-luciferin, potassium salt and 0.5 mM magnesium sulphate were added from filter-sterilized stock solutions. Molten agar with luciferase, luciferin and magnesium sulphate was added to a white 96-well plate (0.2 mL per well) and the plate was left to solidify and dry overnight at room temperature. Tube pre-cultures (3 mL) of *Staphylococcus aureus* ATCC 25923 and *Escherichia coli* ATCC 25922 in nutrient broth were incubated overnight at 37°C and 150 rpm. Dilution series of the pre-cultures were prepared in sterile 25 mM MOPS buffer at pH 7.0, and the highest cell concentration OD_600_ was set to 0.01 (approx. 10^7 CFU/mL). The centre of the surface of solidified agar was inoculated with 2 μL cell suspension per well. Sterile MOPS buffer (2 μL) was added to control wells. The cell concentration of the dilution series was determined by plating 0.05 mL of the appropriate dilution steps on nutrient agar. All outer wells of the 96-well plate were filled with 0.3 mL sterile water to reduce evaporation in wells with agar. After inoculation, the 96-well plate was incubated covered with a transparent lid for 20 h at 37°C in a SpectraMax M5 plate reader and luminescence was measured every 10 min from top.

### Online monitoring of eATP in broth cultures

Extracellular ATP (eATP) released during growth of bacteria in broth culture was analysed by adding 10 μg/mL thermostable luciferase, 0.15 mM D-luciferin and 0.5 mM magnesium sulphate to nutrient broth (from concentrated stock solutions). Supplemented medium was filter-sterilized and preincubated overnight at room temperature to burn-off ATP present in medium. Tube pre-cultures (3 mL) of *Staphylococcus aureus* ATCC 25923 and *Escherichia coli* ATCC 25922 in nutrient broth were incubated overnight at 37°C and 150 rpm. Pre-cultures were diluted in sterile saline (0.9% NaCl) to an OD_600_ of 0.01 (approx. 10^7^ CFU/mL). Wells of white 96-well plate were filled with 0.198 mL medium containing luciferase/luciferin, outer wells were filled with 0.3 mL sterile water to prevent drying out. The plate was prewarmed to 37°C and then inoculated with 2 μL cell suspension (starting cell density approx. 10^5 CFU/mL). Sterile saline was added to control wells. After inoculation, the 96-well plate was incubated covered with a transparent lid for 20 h at 37°C in a SpectraMax M5 plate reader and luminescence was measured every 10 min from top. For comparison, 7.92 mL medium containing luciferase/luciferin were filled to 15 mL glass tubes, prewarmed to 37°C and inoculated with 80 μL of similar cell suspensions (starting cell density approx. 10^5^ CFU/mL). Tubes were incubated at 37°C and 150 rpm and sampled every 20 min for analysis of optical density (in transparent 96-well plate, 0.3 mL per well).

### Quantification of eATP and total ATP in broth cultures

In order to transform luminescence intensity (relative light units, RLU) to ATP concentration in nanomol per liter, a standard curve was measured in nutrient broth supplemented with 10 μg/L thermostable luciferase, 0.15 mM D-luciferin and 0.5 mM magnesium sulfate. The supplemented medium was added to a white 96-well plate (0.2 mL per well) and the plate was pre-incubated with lid for 4 h at 37°C in order to burn-off ATP background from complex medium components. An 1:1 v/v dilution series of ATP was prepared in ultrapure water, ranging from 20 to 0.02 μM. ATP dilutions were added at a ratio of 1:100 v/v (2 μL to 0.2 mL) to supplemented nutrient broth in the 96-well plate using an 8-channel pipet and luminescence was measured immediately in the plate reader (500 msec integration time, similar instrument settings as in online growth assays). RLU values were plotted against final ATP concentrations and the slope of a linear regression curve was used as coefficient (RLU/nM ATP) for transformation of RLU to nM ATP in experiments with cells. In order to determine the ratio of eATP to total ATP in bacterial cultures in 96-well plates, *Staphylococcus aureus* ATCC 25923 and *Escherichia coli* ATCC 25922 were inoculated at 10^5^ CFU/mL to replicate wells with 0.2 mL nutrient broth supplemented with thermostable luciferase, D-luciferin and magnesium sulfate. The plate was incubated with cover at 37°C and luminescence (500 ms integration time, endpoint) in part of the wells (three per strain) was measured after 3 h (eATP). Then, 0.1% dodecyltrimethylammonium bromide (lysis agent) was added from a 40-fold concentrated stock solution (4.1% in water, 5 μL to 0.2 mL) and luminescence was measured again after 5 min lysis time (total ATP). The same procedure was applied to the remaining wells with growing bacteria after 5 h incubation time. eATP and total ATP concentrations were calculated using the RLU/nM ATP coefficient derived from the ATP standard curve determined under the same conditions (S1 Fig).

### Viable cell count based on luminescence onset time in broth culture

Nutrient broth was supplemented with 10 μg/mL thermostable luciferase, 0.4 mM D-luciferin and 1 mM MgCl_2_. Supplemented medium was sterilized by filtration (0.2 μm) and incubated for 2.5 h at 30°C in order to burn-off ATP background from complex nutrients. Wells of a white 96-well plate were filled with 0.198 mL medium containing luciferase/luciferin. Overnight agar plate cultures of *Pseudomonas fluorescens* ATCC 49838, *Pantoea agglomerans* RKI 16–2, *Bacillus cereus* ATCC 14579, *Staphylococcus aureus* ATCC 29213 and *Escherichia coli* ATCC 25922 were resuspended in sterile phosphate buffered saline, serially diluted in the same diluent and then inoculated to the microplate (2 μl per well). An optical density (OD, 600 nm) of 0.1 was considered to represent a cell concentration of 10^8^ CFU/mL for all strains. The microplate was covered with a transparent lid and incubated at 30°C in a SpectraMax M5 plate reader, relative light units (RLU) were measured automatically every 10 min. In a subsequent experiment, equal volumes of cell suspensions (OD 0.1) of all five stains were mixed, serially diluted in sterile PBS and then inoculated to a similar microplate (2 μl per well, 3 replicate mixtures and dilution series). Cell concentration of mixtures were determined by plating on tryptic soy agar. Luminescence onset times were deduced from the time course of luminescence (RLU) for each well separately as follows: time from start of incubation when (i) rate of luminescence increase for four consecutive time points reached values higher than 150 RLU/h, (ii) rate of luminescence increase did not drop below 100 RLU/h for the next three time points. Subsequently, the onset time deduced from raw RLU time course data was plotted against initial cell concentration in log scale, and the regression coefficient and formula of a fitted exponential function were calculated in Excel.

### eATP online assay for analysis of antimicrobial agents

Nutrient broth was supplemented with 10 μg/mL thermostable luciferase, 0.15 mM D-luciferin and 0.5 mM magnesium sulfate, filter-sterilized and pre-incubated overnight at room temperature. Wells of a white 96-well plate were filled with 0.196 mL supplemented medium and incubated with lid for another 2.5 h at 37°C to burn-off medium ATP background, outer wells were filled with 0.3 mL sterile water to reduce evaporation in inner wells. Antibiotic resistant *E*. *coli* RKI 66/09 (*ampC*) and antibiotic sensitive *E*. *coli* ATCC 25922 were cultivated for 20 h at 37°C and 150 rpm in nutrient broth and then diluted to an OD600 of 0.01 in sterile PBS. Wells were inoculated with 2 μL diluted cell suspension, resulting in a starting cell density of approx. 10^5^ CFU/mL. The plate was incubated with transparent lid for 1 h at 37°C in the plate reader and luminescence was measured every 2 min from top. Then 4 μg/mL Imipenem, 50 μg/mL Ampicillin or 50 μg/mL Polymyxin B was added from 100-fold concentrated stock solutions in water, no antimicrobial agents were added to control wells. Incubation at 37°C and luminescence measurements were continued for another three hours in the plate reader.

In a second experiment, nutrient broth pH 7.4 (5 g/l peptone, 5 g/l NaCl, 2 g/l yeast extract, 1 g/l meat extract) was autoclaved and cooled to room temperature, then 10 μg/mL thermostable luciferase, 0.4 mM D-luciferin and 1 mM MgCl_2_ were added from concentrated stock solutions. Supplemented medium was sterilized by filtration (0.2 μm), pre-incubated for 2.5 h at 30°C and then added to a white 96-well plate (0.19 mL per well). Ampicillin was added from a dilution series of filter-sterilized stock solution prepared in sterile ultrapure water (5 μL per well). Sterile water was added to control wells without antibiotic. Wells were inoculated with 5 μl of cell suspensions (OD_600_ 0.1, prepared in sterile PBS from agar plate pre-cultures) of either antibiotic resistant or antibiotic sensitive *E*. *coli*. Initial cell concentration in wells was approximately 2.5*10^6^ CFU/mL. The 96-well plate was incubated at 37°C in a luminescence microplate reader and luminescence (RLU) was measured every 2 min for 3 h. Incubation at 37°C was continued for in total 15 h, then optical density (600 nm) of all wells was measured after transfer of the liquid to a transparent 96-well plate.

In order to reduce ATP background from complex medium components and enhance ATP generation, a low complex nutrient medium with easy metabolizable carbon and energy sources was designed. The medium contained 20 mM MOPS buffer, 0.05 g/L KH_2_PO_4_, 0.5 g/L glucose, 0.5 g/L glycerol, 0.1 g/L sodium pyruvate, 0.1 g/L yeast extract, 0.05 g/L peptone, 0.05 g/L tryptone, 0.1 mM CaCl_2_ and 0.5 mM MgSO_4_. After setting pH to 7.0 with 1 N NaOH, 10 μg/L thermostable luciferase and 0.15 mM D-luciferin were added from stock solutions and the supplemented medium was filter-sterilized. The medium was filled into a white 96-well plate (0.196 mL per well, outer wells filled with 0.3 mL sterile water) and 2 μL filter-sterilized antibiotic stock solution in water or sterile water was added. Final concentration of Imipenem was 4 μg/mL and final concentration of Ampicillin was 50 μg/mL. The plate with supplemented medium was pre-incubated with transparent lid for 4 h at 37°C in order to bring background to lowest possible level. Antibiotic resistant *E*. *coli* RKI 66/09 (*ampC*) and antibiotic sensitive *E*. *coli* ATCC 25922 were cultivated for 22 h at 37°C and 150 rpm in nutrient broth and then diluted to an OD_600_ of 0.1 in sterile PBS. Wells were inoculated with 2 μL cell suspension (initial cell concentration approx. 1·10^6^ CFU/mL) and luminescence was measured for 2 h every 2 min in a SpectraMax M5 plate reader at 37°C.

## Supporting information

S1 Fig ATP standard curve in nutrient broth supplemented with 10 μg/mL thermostable luciferase, 0.15 mM D-luciferin and 0.5 mM magnesium sulfate. Endpoint measurements in white 96-well plate immediately after addition of ATP in SpectraMax M5 plate reader, total assay volume was 0.2 mL. Relative light units (RLU) were corrected for average RLU of control wells without added ATP (medium background). Trendline represents linear regression by least squares method, equation for linear relationship and correlation coefficient is shown in plot area.
(TIF)

S2 Fig Effect of 4 μg/mL Imipenem, 50 μg/mL Ampicillin and 50 μg/mL Polymyxin B on growth of antibiotic sensitive *Escherichia coli* ATCC 25922 and antibiotic resistant *Escherichia coli* RKI 66/09 AmpC (CMY-2). Bacterial strains were cultivated in MOPS-buffered, low complex nutrient medium (similar medium as in Fig 7) in 15 mL glass tubes with 2 mL liquid volume. Antibiotics were added from 100-fold concentrated stock solutions, sterile water was added to positive control cultures. Tubes were inoculated 1:1000 v/v from pre-cultures grown in nutrient broth and incubated for 17 h at 37°C and 150 rpm. Optical density was measured in a transparent 96-well plate (0.3 mL sample volume) and blank OD_600_ value of the same volume of sterile medium was subtracted. Closed bars: antibiotic sensitive *E*. *coli*, open bars: antibiotic resistant *E*. *coli*.
(TIF)

S1 Data (XLSX)

S2 Data (XLSX)

S3 Data (XLSX)

S4 Data (XLSX)

S5 Data (XLSX)
