# Supplementary figures and images for: Real-time monitoring of extracellular ATP in bacterial cultures using thermostable luciferase
Source: PLoS One. 2021 Jan 22;16(1):e0244200. doi: 10.1371/journal.pone.0244200 (PMC7822345; doi:10.1371/journal.pone.0244200)

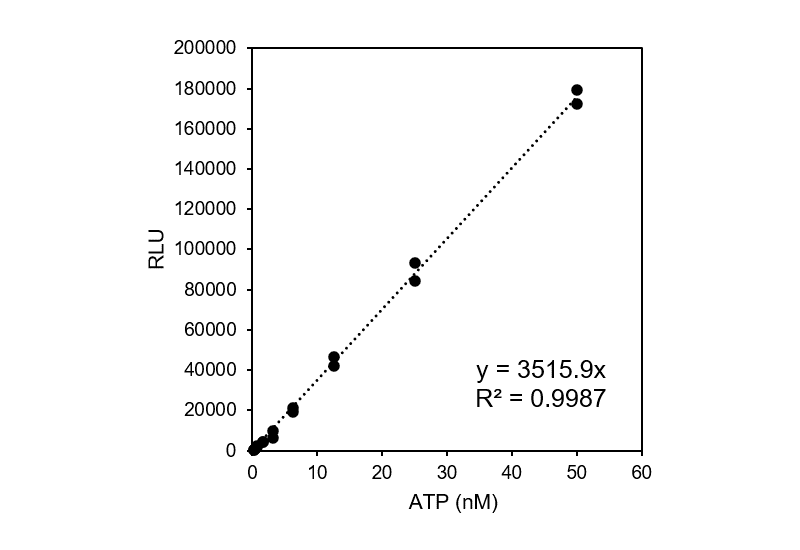

Supplement: S1 Fig — Endpoint measurements in white 96-well plate immediately after addition of ATP in SpectraMax M5 plate reader, total assay volume was 0.2 mL. Relative light units (RLU) were corrected for average RLU of control wells without added ATP (medium background). Trendline represents linear regression by least squares method, equation for linear relationship and correlation coefficient is shown in plot area. (TIF) [file pone.0244200.s001.tif]

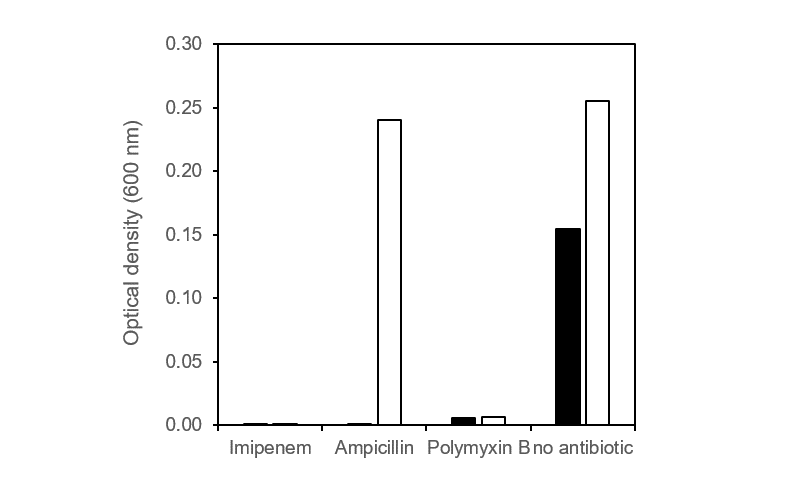

Supplement: S2 Fig — Bacterial strains were cultivated in MOPS-buffered, low complex nutrient medium (similar medium as in Fig 7) in 15 mL glass tubes with 2 mL liquid volume. Antibiotics were added from 100-fold concentrated stock solutions, sterile water was added to positive control cultures. Tubes were inoculated 1:1000 v/v from pre-cultures grown in nutrient broth and incubated for 17 h at 37°C and 150 rpm. Optical density was measured in a transparent 96-well plate (0.3 mL sample volume) and blank OD600 value of the same volume of sterile medium was subtracted. Closed bars: antibiotic sensitive E. coli, open bars: antibiotic resistant E. coli. (TIF) [file pone.0244200.s002.tif]
